# Co-Fermentation of *Wickerhamomyces anomalus* and *Saccharomyces cerevisiae* in Bread Dough: Effects on Ethyl Acetate Production and Flavor Enhancement

**DOI:** 10.4014/jmb.2604.04026

**Published:** 2026-07-30

**Authors:** Jeong-Ah Yoon, Young Ho Seo, Myoung-Dong Kim

**Affiliations:** 1 Institute of Fermentation and Brewing, Kangwon National University, Chuncheon 24341, Republic of Korea; 2 Department of Mechanical Convergence Engineering, Kangwon National University, Chuncheon 24341, Republic of Korea; 3 Department of Food Science and Biotechnology, Kangwon National University, Chuncheon 24341, Republic of Korea

**Keywords:** Bread, *Saccharomyces cerevisiae*, *Wickerhamomyces anomalus*, Flavor, Fermentation

## Abstract

Fermentation affects the quality of bakery products. *Saccharomyces cerevisiae* is the traditional baking yeast, but the desire for alternative flavor profiles has driven interest in non-*Saccharomyces* yeasts. *Wickerhamomyces anomalus*, a non-*Saccharomyces* yeast with high ester-producing ability, has attracted attention as a promising co-fermentation partner. Although previous studies have explored its co-fermentation potential in alcoholic beverage production, the effects of its co-fermentation with *S. cerevisiae* on dough fermentation characteristics, bread quality, and flavor remain largely unexplored. The aim of this study was to evaluate the baking potential of *W. anomalus* strains isolated in Korean nuruk and to examine the effects of co-fermentation with *S. cerevisiae* on bread quality. Dough co-fermented with both yeasts showed improved leavening ability and distinct volatile compound profiles compared with single-fermented dough. Specifically, ethyl acetate levels were higher in co-fermented dough than in dough fermented only with *S. cerevisiae*. Among the strains tested, *W. anomalus* NIYL25845 exhibited gas production comparable to the control strain KCTC27761 and the highest ethyl acetate production during co-fermentation. Bread co-fermented with *S. cerevisiae* and *W. anomalus* NIYL25845 had a similar specific volume and texture to those of bread fermented with *S. cerevisiae* alone, although the co-fermented bread exhibited higher baking loss. The levels of esters and higher alcohols increased compared with the control (sole *S. cerevisiae* fermentation), and the ethyl acetate content reached 3.80 ± 0.54 ppm — the highest among all bread samples tested. These results suggest that co-fermentation with *W. anomalus* NIYL25845 is a promising strategy for enhancing bread flavor without compromising bread quality.

## Introduction

Fermentation influences loaf volume, crumb texture, and flavor and is crucial to overall product quality in the baking industry. Traditionally, *Saccharomyces cerevisiae* has been the most common yeast used for fermentation because of its strong leavening ability and reliable performance in industrial baking processes [1]. However, the continued reliance on a single yeast species has raised concerns about the uniformity of flavor profiles [2].

Non-*Saccharomyces* yeasts have attracted interest for their unique metabolic traits, which can diversify the flavor profiles of fermented foods. Among these yeasts, *Wickerhamomyces anomalus* is recognized for its high osmotolerance [3] and capacity to produce esters such as ethyl acetate, phenylethyl acetate, and ethyl propionate [4]. Ethyl acetate is one of the major esters produced by *W. anomalus* and is known to impart fruity aroma notes that enhance the sensory quality of fermented foods [5-7]. These characteristics make *W. anomalus* a promising yeast for flavor enhancement in a wide range of food fermentations, including breadmaking.

Several studies have linked its presence to increased ester production and enhanced sensory qualities in baijiu, cider, and wine fermentation [8-11]. However, many non-*Saccharomyces* yeasts have limited fermentation capacity when used as sole starters, a challenge that has prompted the development of co-fermentation strategies with *S. cerevisiae* [12-14].

Co-fermentation approaches are regarded as effective methods for maintaining the strong fermentative performance of *S. cerevisiae* while also promoting the expression of the distinctive flavor profiles of non-*Saccharomyces* yeasts [12, 13, 15]. Indeed, co-fermentation of *W. anomalus* with *S. cerevisiae* has been shown to enhance ester-driven aroma diversity and improve sensory quality in fruit wine fermentation, such as in mulberry wine and cider [16, 17]. However, research on co-fermentation with non-*Saccharomyces* yeasts, such as *W. anomalus*, has mainly focused on fermented beverages, whereas the potential roles of these yeasts in the baking industry remain largely unexplored. Bread fermentation, unlike beverage fermentation, involves gluten network formation, gas production and retention, and the loss of volatile compounds during baking — all of which collectively influence final bread quality [18]. Therefore, it remains unclear whether the metabolic interactions observed during co-fermentation in fermented beverages can be translated to bread fermentation and ultimately contribute to aroma enhancement after baking.

The aim of this study was to assess the potential of *W. anomalus* strains isolated in Korea to enhance flavor during co-fermentation with *S. cerevisiae* in breadmaking. To achieve this, specific growth rate, leavening ability, volatile compound profiles, and bread quality were analyzed. This research provides valuable data for developing alternative yeast starters that diversify flavor profiles and enhance the overall quality of baked goods.

## Materials and Methods

### Strain and Cultivation Conditions

*W. anomalus* strains isolated in Korea were used, and *S. cerevisiae* Saf-instant^®^ Red (Lesaffre, France) and *W. anomalus* KCTC27761 (Korean Collection for Type Cultures, Republic of Korea) were used as control strains (Table S1). All strains were preserved at -80°C in 20% (v/v) glycerol stock. Before the experiments, the strains were activated by incubation in YEPD broth (10 g/L yeast extract, 20 g/L peptone, 20 g/L glucose) at 30°C for 24 h. The cells were harvested by centrifugation (5,000 × *g*, 5 min), washed twice with distilled water, and resuspended in distilled water to obtain a cell suspension for subsequent experiments.

### Evaluation of Growth and Gas Production

The liquid dough (LD) medium used in this study was based on a dough-simulating medium described previously [19, 20], with ingredient concentrations adjusted to meet the specific objectives of the experiment.

To evaluate the growth of *W. anomalus* under baking-related conditions, pre-cultured cells were inoculated into 200 μL of LD broth to an OD_600_ of 0.1, and then incubated at 30°C for 24 h. Growth curves were generated from OD_600_ measurements using a LogPhase 600 (Agilent Technologies, USA).

For gas production analysis, each strain was inoculated into 200 mL of LD broth at an OD_600_ of 1.0, and gas production was measured every 20 min at 30°C for 6 h using a Fermograph III (Atto, Japan).

### Assessment of Hemolytic Activity

Cell suspensions were washed with sterile distilled water, adjusted to an OD_600_ of 0.1, and spotted in 20 μL aliquots onto Columbia agar base (MBcell, Republic of Korea) supplemented with 5% (v/v) sheep blood (MBcell). The plates were incubated at 30°C for 24 h. Hemolytic activity was evaluated by observing hemolysis zones. The formation of a greenish zone was interpreted as α-hemolysis, a clear zone as β-hemolysis, and the absence of hemolysis as γ-hemolysis [21].

### Dough Preparation and Baking

Dough preparation and baking were based on a milk bread recipe [22], with modifications. Dough was prepared with *S. cerevisiae* and *W. anomalus* at various inoculation ratios (*S. cerevisiae*: *W. anomalus* = 0.5:0, 1:0, 0.5:0.5, 0:0.5, and 0:1).

Kneading was performed for 10 min using a dough kneader (KVL4100S; Kenwood, UK). Unsalted butter (Anchor, New Zealand) was then added, followed by an additional 5 min of kneading.

The dough underwent primary fermentation at 27°C and 80% relative humidity for 1 h, after which it was divided into three 170 g portions, shaped into three-lobed forms, and placed in loaf pans. A second fermentation was performed at 38°C and 80% relative humidity for 1 h. Baking was performed in an oven (XF003-K; UNOX, Italy) at 170°C for 20 min. The loaves were cooled at room temperature for 30 min before analysis.

### Dough Characterization

**Assessment of leavening ability.** Leavening ability was evaluated using 30 g of dough in a Fermograph III (Atto) at 30°C, with gas production measured every 20 min for 6 h.

**Volatile compound profiling.** The volatile compound profile was analyzed using an electronic nose (E-nose; Heracles NEO; Alpha MOS, France). Two-gram portions of dough fermented at 30°C for 4 h were placed into 20 mL headspace vials (Agilent Technologies). To equilibrate the headspace with volatile compounds, the samples were shaken at 40°C for 30 min, and 5 mL of the headspace gas was then injected into the E-nose. Volatile compounds were separated on two columns with different polarities (MXT-5: 10 m × 0.18 mm × 0.4 μm; MXT-1701: 10 m × 0.18 mm × 0.4 μm; Restek, USA) and detected with flame ionization detectors (FIDs). The oven temperature was initially held at 40°C for 2 s, increased to 80°C at 1°C/s, subsequently increased to 250°C at 2°C/s, and then held at 250°C for 21 s. Hydrogen was used as the carrier gas at a constant flow rate of 1 mL/min. Volatile compounds were identified by comparing their Kovats retention indices [23, 24] with those in the AroChemBase library (v2020; Alpha MOS).

### Bread Characterization

**Determination of baking loss and specific volume.** Baking loss was calculated from the dough weight before baking (A) and loaf weight after baking (B), as follows [25]:

Baking loss (%) = [(A − B) / A] × 100

The loaf volume (cm^3^) was measured using the rapeseed displacement method (AACC Method 10−05.01) [26]. Specific volume was calculated as loaf volume divided by loaf weight (g).

**Color Analysis.** The colors of the crust and crumb were each measured using a colorimeter (CR-20; Konica Minolta, Japan). Lightness (L*), redness (a*), and yellowness (b*) were measured, and the total color difference (Δ*E*) was calculated using the following equation [27]:

Δ*E* = √((ΔL*)^2^ + (Δa*)^2^ + (Δb*)^2^)

where ΔL*, Δa*, and Δb* represent the differences between bread fermented only with *S. cerevisiae* and bread co-fermented with *S. cerevisiae* and *W. anomalus*.

**Texture Analysis.** Bread texture was measured using a texture analyzer (DO736250; ZwickRoell Group, Germany). Bread samples were sliced to a thickness of 2.5 cm and compressed twice with a cylindrical flat probe (36 mm diameter) at a test speed of 400 mm/min. Hardness (N) was defined as the maximum force during the initial compression cycle. Springiness was defined as the ratio of the height recovered between the end of the first compression and the start of the second compression to the original sample height. Cohesiveness was defined as the ratio of the area under the second compression curve to that under the first compression curve, and chewiness (N) was defined as the product of hardness, cohesiveness, and springiness.

**Volatile Compound Analysis.** For volatile compound analysis, samples were analyzed using gas chromatography–mass spectrometry (GC–MS; 8890 GC/5975 MSD; Agilent Technologies). Bread (2 g) was placed in a 20 mL headspace vial (Agilent Technologies). To equilibrate the headspace with volatile compounds, the vials were shaken at 60°C for 20 min. Solid-phase microextraction (SPME) was performed using a 65 μm Supelco PDMS/DVB fiber (MilliporeSigma, USA), and the extracted volatiles were injected into the 8890 GC system. Helium was used as the carrier gas at a flow rate of 1.0 mL/min. Volatile compounds were separated on a DB-WAX column (30 m × 0.25 mm × 0.25 μm; Agilent Technologies). The oven temperature was initially held at 40°C for 5 min, then increased to 150°C at a rate of 2°C/min and held at 150°C for 10 min. Quantification was performed in the selected-ion monitoring (SIM) mode. Concentrations of volatile compounds were determined using calibration curves derived from reference standards.

### Statistical Analysis

All experiments were performed in triplicate or quintuplicate. Data are presented as means ± standard deviation (SD). Statistical analyses were performed using SPSS Statistics 26 (IBM Corp., USA), with *p* < 0.05 considered statistically significant. Statistical differences among groups were analyzed using one-way analysis of variance (ANOVA) followed by Duncan’s multiple-range test [28]. Multivariate statistical analyses, including principal component analysis (PCA), permutational multivariate analysis of variance (PERMANOVA), and partial least squares discriminant analysis (PLS-DA), were conducted using MetaboAnalyst 6.0 [29].

## Results

### Strain Selection

The growth characteristics of *W. anomalus* strains under simulated dough conditions were assessed by measuring the specific growth rate in LD broth (Fig. S1). All *W. anomalus* strains except for NIYL30536 showed higher growth rates than that of *S. cerevisiae* Saf-instant. Among these, five strains with higher growth rates than that of the control strain, *W. anomalus* KCTC27761, were selected for further experiments.

Total gas production in LD broth by *W. anomalus* strains was compared (Fig. S2). *S. cerevisiae* Saf-instant exhibited significantly higher gas production levels than those of *W. anomalus* strains. Of the five strains selected based on growth rate, *W. anomalus* NIYL11942, NIYL11971, and NIYL25845 produced gas at levels comparable to that of the control strain, *W. anomalus* KCTC27761. Additionally, the three *W. anomalus* strains showed γ-hemolysis on blood agar (Fig. S3) and were therefore selected for further experiments.

### Leavening Ability

The leavening ability of dough inoculated with *S. cerevisiae* and *W. anomalus* at different ratios was assessed by measuring gas production (Table 1). For all strains, dough inoculated at a 0:1 ratio produced more gas than that inoculated at a 0:0.5 ratio. Gas production in dough fermented solely with *W. anomalus* was generally lower than that in dough fermented with *S. cerevisiae* Saf-instant. For dough fermented solely with *W. anomalus*, NIYL25845 showed the highest gas production at both inoculation ratios. Dough co-fermented with *S. cerevisiae* and *W. anomalus* at a 0.5:0.5 ratio produced more gas than dough fermented solely with *S. cerevisiae*. Notably, dough co-fermented with NIYL11971 or NIYL11942 showed the highest gas production levels, reaching 306.32 ± 4.39 mL and 298.89 ± 0.98 mL, respectively. Dough co-fermented with NIYL25845 generated 280.57 ± 3.16 mL of gas, which was comparable to dough co-fermented with KCTC27761 (control strain).

### Volatile Compound Profiles

**Fig. 1:**
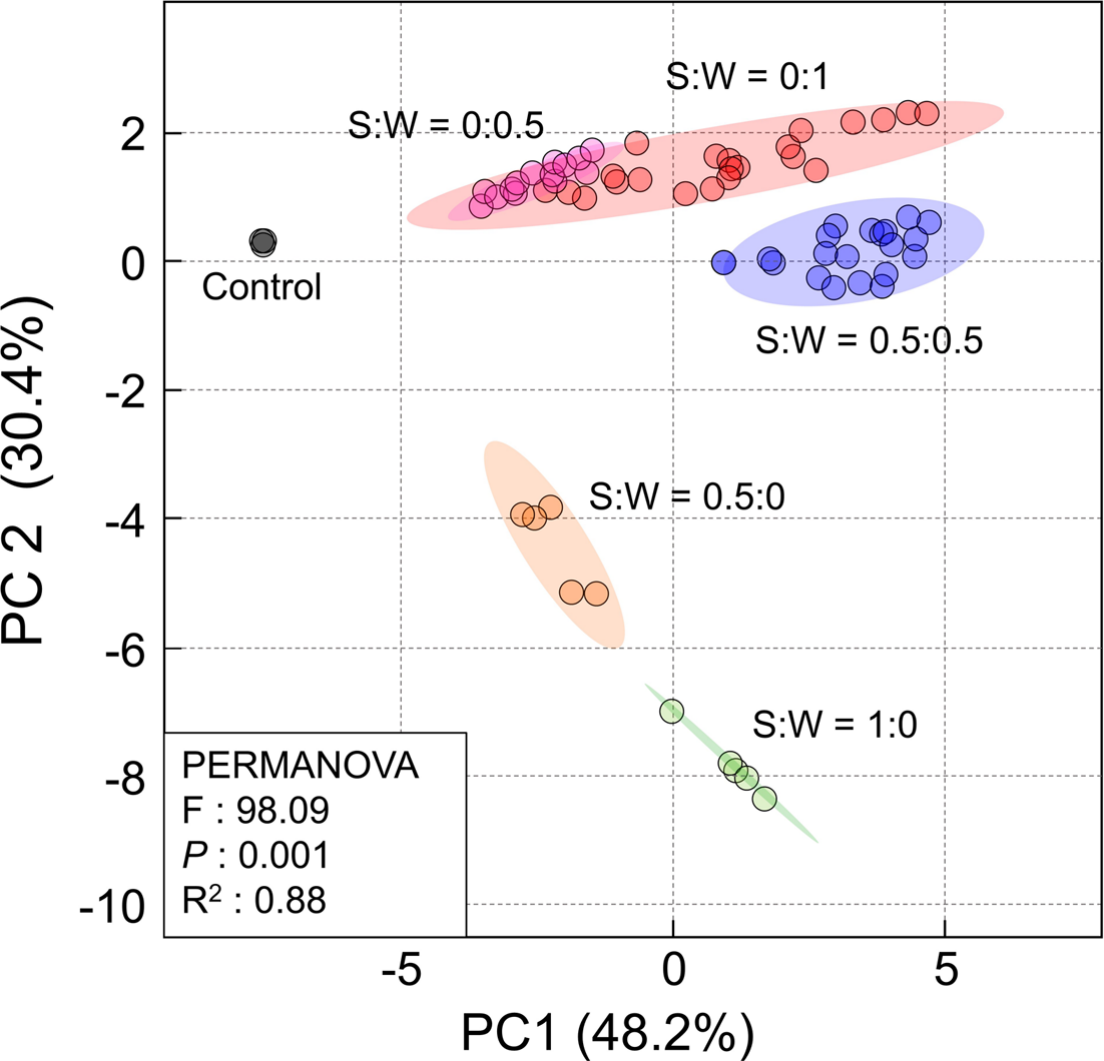
Principal component analysis score plot of volatile compounds in dough fermented under various conditions. Control refers to an uninoculated dough, and S:W indicates the inoculation ratio of *Saccharomyces cerevisiae* Saf-instant to *Wickerhamomyces anomalus*. The difference between groups was assessed using permutational multivariate analysis of variance (PERMANOVA).

**Fig. 2:**
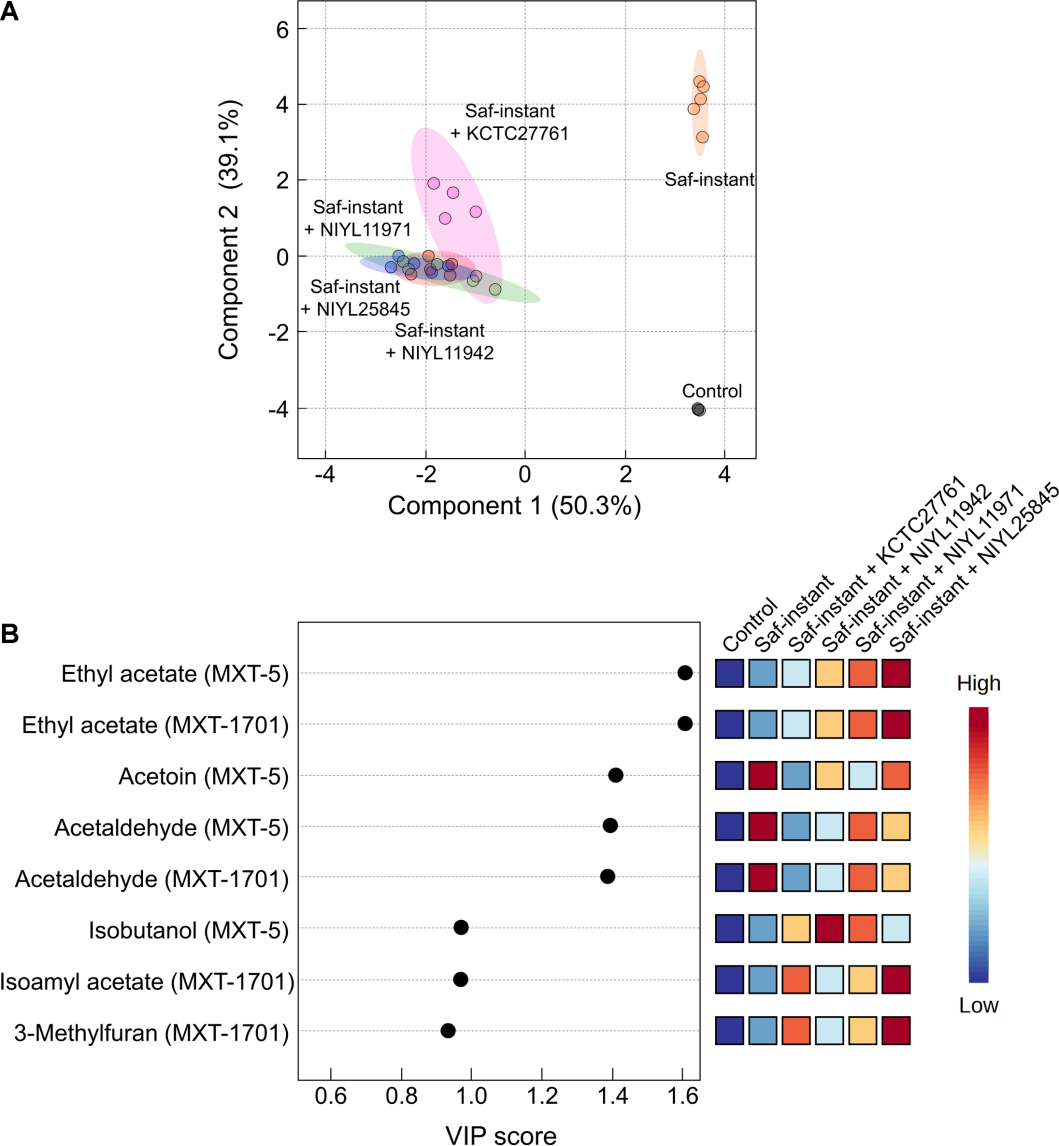
Partial least squares discriminant analysis score plot (A) and variable importance in projection (VIP) scores (B) for volatile compounds in dough fermented with *Saccharomyces cerevisiae* Saf-instant and/or *Wickerhamomyces anomalus* strains. Control refers to an uninoculated dough. Parentheses on the y-axis of panel B denote the electronic nose column used to detect each volatile compound.

PCA and PLS-DA were performed on volatile compound data obtained using an E-nose (Figs. 1 and 2). Dough fermented solely with *S. cerevisiae* was distributed in the negative region of PC2, while dough fermented solely with *W. anomalus* was in the positive region (Fig. 1). In both the *S. cerevisiae* and *W. anomalus* groups, dough with higher inoculation levels was distributed in the positive region of PC1; whereas, co-fermented dough was spread between the two groups.

PLS-DA analysis of dough fermented solely with *S. cerevisiae* or co-fermented with *W. anomalus* demonstrated that the two groups were distinct from each other (Fig. 2A). Additionally, co-fermented dough showed distinct distribution patterns according to the *W. anomalus* strain used. Variable importance in projection (VIP) scores showed that volatile compounds such as ethyl acetate, acetoin, acetaldehyde, isobutanol, and isoamyl acetate helped differentiate the dough samples (Fig. 2B). The level of ethyl acetate was higher in co-fermented dough than in dough fermented solely with *S. cerevisiae*, and co-fermentation with NIYL25845 produced the highest level. In contrast, acetoin and acetaldehyde were present at higher levels in dough fermented solely with *S. cerevisiae*.

### Evaluation of Baking Performance

Baking performance was evaluated under representative single-fermentation and co-fermentation conditions, using *S. cerevisiae*:*W. anomalus* ratios of 1:0, 0:1, and 0.5:0.5.

**Fig. 3:**
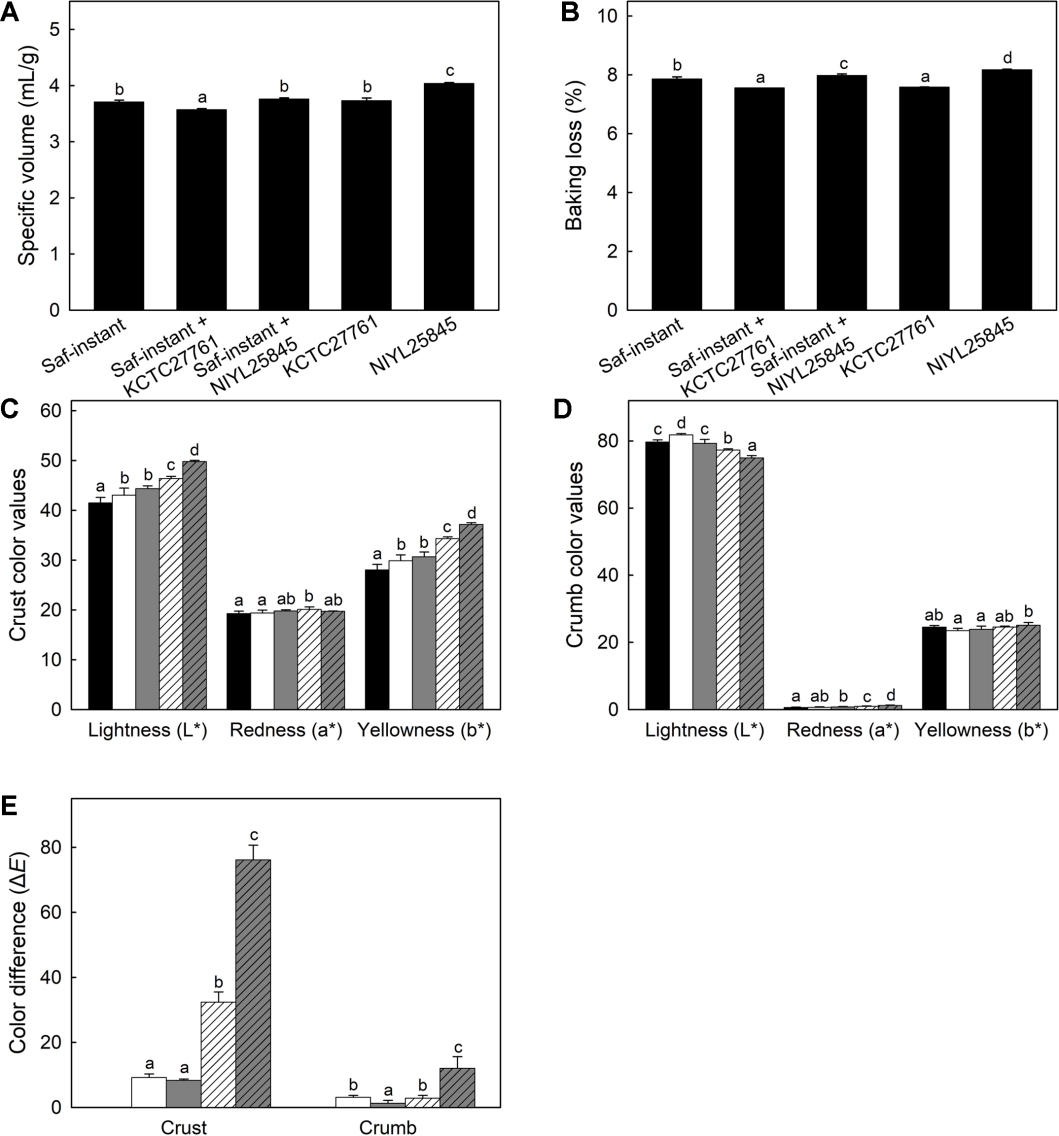
Specific volume (A), baking loss (B), crust color (C), crumb color (D), and color difference (E) of bread prepared with *Saccharomyces cerevisiae* Saf-instant and *Wickerhamomyces anomalus*. In panels C–E, the black, white, gray, light hatched, and dark hatched bars represent Safinstant, Saf-instant + KCTC27761, Saf-instant + NIYL25845, KCTC27761, and NIYL25845, respectively. Color difference (Δ*E*) was calculated relative to the bread made with *S. cerevisiae* Saf-instant. Different letters indicate significant differences among groups (*p* < 0.05).

Bread fermented solely with NIYL25845 showed the highest specific volume (4.04 ± 0.02 mL/g) among all groups tested (Fig. 3A).

The specific volume of bread co-fermented with *S. cerevisiae* and NIYL25845 was 3.76 ± 0.02 mL/g, comparable to that of bread fermented solely with *S. cerevisiae*. However, the baking loss of bread fermented solely with NIYL25845 was the highest among all bread samples tested, reaching 8.18 ± 0.02% (Fig. 3B). Bread co-fermented with *S. cerevisiae* and NIYL25845 exhibited the second-highest baking loss, while both the single- and co-fermented breads containing KCTC27761 showed the lowest baking loss. In the crust, L* and b* were highest in bread fermented solely with NIYL25845, with higher values also observed in the other *W. anomalus*-fermented breads compared with bread fermented solely with *S. cerevisiae* (Fig. 3C). In contrast, a* showed only minor differences among all bread samples. In the crumb, L* was highest in bread co-fermented with KCTC27761 and lowest in bread fermented solely with NIYL25845, and a* was highest in bread fermented solely with NIYL25845; b* showed only minor differences among all bread samples (Fig. 3D). The ΔE values were higher in the crust than in the crumb (Fig. 3E). Breads fermented solely with *W. anomalus* showed higher Δ*E* values than co-fermented breads, with bread fermented solely with NIYL25845 exhibiting the highest ΔE values — 76.13 ± 4.55 in the crust and 12.05 ± 3.53 in the crumb.

The different fermentation groups showed no significant differences in hardness, springiness, or chewiness (Table 2).

**Fig. 4:**
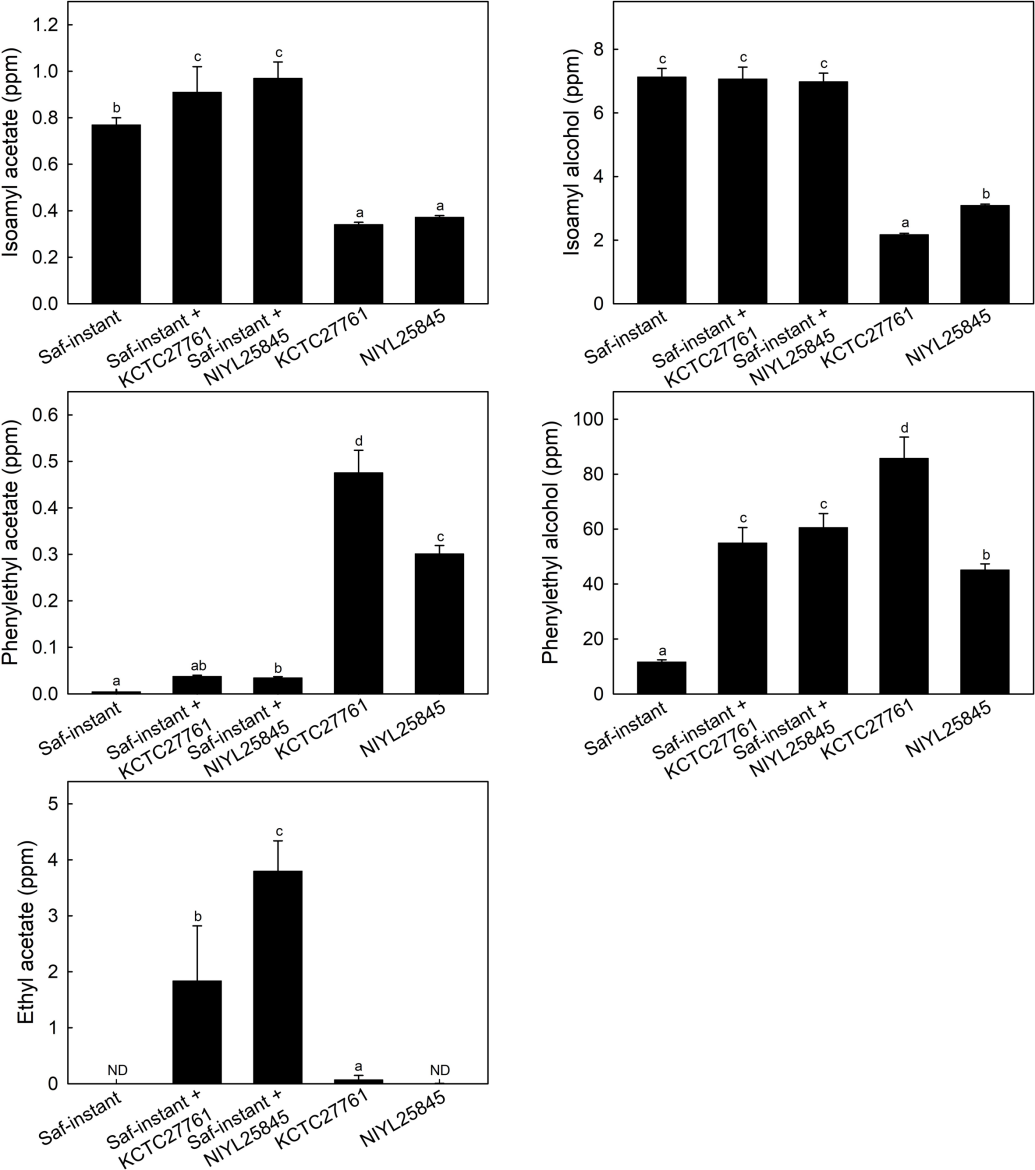
Volatile compounds in bread fermented with *Saccharomyces cerevisiae* Saf-instant and *Wickerhamomyces anomalus*. Data are presented as means ± standard deviation of three independent experiments. Different letters denote statistically significant differences (*p* < 0.05).

Cohesiveness was lower in bread fermented solely with *W. anomalus* than in bread fermented solely with *S. cerevisiae* or in co-fermented bread. Analysis of volatile compounds revealed that co-fermented breads contained higher levels of isoamyl acetate and ethyl acetate than bread fermented solely with *S. cerevisiae*, with bread co-fermented with NIYL25845 exhibiting the highest ethyl acetate concentration (3.80 ± 0.54 ppm) among all samples (Fig. 4). In contrast, bread fermented solely with *W. anomalus* contained lower levels of isoamyl acetate, isoamyl alcohol, and ethyl acetate, but higher levels of phenylethyl acetate and phenylethyl alcohol than the other bread samples.

## Discussion

This study evaluated the baking potential of *W. anomalus* strains isolated in Korea and investigated the effects of co-fermentation with *S. cerevisiae* on fermentation characteristics and bread quality.

Three *W. anomalus* strains showing equal or better performance than that of *W. anomalus* KCTC27761 in both specific growth rate and gas production in LD broth were selected. These strains did not exhibit hemolysis on blood agar, indicating no hemolysis-related safety concerns and supporting their potential applicability in baking processes. Most *W. anomalus* strains showed higher growth rates, but their gas production was lower than that of *S. cerevisiae*. This indicates that *S. cerevisiae* mainly channels carbon flux toward CO_2_-producing fermentative metabolism through the Crabtree effect, while non-*Saccharomyces* yeasts tend toward more respiratory-dominant metabolic profiles [5, 30].

In dough fermented solely with *W. anomalus*, gas production was lower than that in dough fermented solely with *S. cerevisiae*, which aligns with the growth characteristics observed earlier. However, dough co-fermented with *S. cerevisiae* and *W. anomalus* showed an overall increase in gas production compared with dough fermented with a single strain, indicating improved leavening. Moreover, strain-dependent differences in gas production were observed in co-fermented dough, suggesting that the impact of co-fermentation may depend on the specific strain.

PCA of volatile compound profiles in dough showed that fermentation with either *S. cerevisiae* or *W. anomalus* only produced distinct flavor profiles. Co-fermented dough was more similar in volatile compound profile to dough fermented only with *W. anomalus*, indicating a relatively greater contribution of *W. anomalus* to flavor development during co-fermentation. PLS-DA analysis also showed a distinction between dough fermented solely with *S. cerevisiae* and co-fermented dough. Ethyl acetate, acetoin, and acetaldehyde, each with VIP scores over 1.0 [31], were identified as contributors to the overall distribution pattern in dough. In particular, the ethyl acetate level was higher in co-fermented dough than in dough fermented solely with *S. cerevisiae*, suggesting that the ester-producing metabolic characteristics of *W. anomalus* [14] may have contributed to the enhanced ethyl acetate formation.

This is consistent with previous studies [4, 6, 9] showing that co-fermentation of *S. cerevisiae* and *W. anomalus* enhances the flavor of wine and baijiu and increases the production of ester compounds, including ethyl acetate.

The increased ethyl acetate observed during co-fermentation may be attributable to the utilization of ethanol produced by *S. cerevisiae* as a substrate for ethyl acetate biosynthesis by *W. anomalus*, potentially via alcohol acetyltransferase (ATF) activity [32]. However, as neither enzyme activity nor the underlying metabolic pathway was investigated in the present study, the precise mechanism responsible for the enhanced ethyl acetate production during co-fermentation remains to be elucidated.

In contrast, acetoin and acetaldehyde levels were relatively higher in dough fermented solely with *S. cerevisiae*, indicating that their formation may be attributable to the metabolic activity of *S. cerevisiae*. The reduced levels of these compounds during co-fermentation may be associated with changes in metabolic flux resulting from substrate competition between the two yeast species, and similar metabolic interactions have been reported during co-fermentation of *W. anomalus* and *S. cerevisiae* [33]. However, as these interactions were not directly examined in the present study, the underlying mechanism remains to be elucidated.

These results show that co-fermentation creates a unique flavor profile through metabolic interactions between the two yeast species, highlighting the role of strain combinations in enhancing bread flavor. Ethyl acetate is widely recognized as a fruity ester, and in the present study, it was identified as a representative compound reflecting the ester-producing characteristics of *W. anomalus*. Therefore, ethyl acetate production was used as a major criterion for strain selection.

In comparison with the other co-fermented doughs, dough co-fermented with NIYL25845 yielded gas levels comparable to those of the control strain KCTC27761, while exhibiting the highest ethyl acetate production. Therefore, NIYL25845 was chosen for baking performance testing, because it demonstrated a favorable balance between leavening ability and flavor traits.

Despite the increased gas production in co-fermented dough, bread co-fermented with NIYL25845 had a specific volume similar to that of bread fermented only with *S. cerevisiae*. In contrast, bread fermented solely with *W. anomalus* exhibited a relatively high specific volume despite its low leavening ability in dough. These results suggest that the final loaf volume is influenced not only by gas production during dough fermentation but also by structural changes during baking [34]. Bread fermented with NIYL25845, either in single- or co-fermentation, exhibited higher baking loss than bread fermented solely with *S. cerevisiae*. *W. anomalus* has been reported to possess proteolytic activity capable of hydrolyzing gliadin [35]; the resulting structural changes in the gluten network may have facilitated greater moisture and volatile compound loss during baking [36], thereby contributing to the higher baking loss observed in bread fermented with NIYL25845. However, further studies are needed to clarify the underlying mechanism.

The crust of bread fermented with *W. anomalus* showed higher L* and b* values than did the crust of bread fermented only with *S. cerevisiae*. This may be due to a relative decrease in Maillard reactions and caramelization resulting from lower levels of residual reducing sugars [37]. In contrast, the changes in L* and a* values observed in the crumb may be linked to differences in microstructure and moisture distribution rather than browning reactions [38]. The textural properties of the single- and co-fermented bread were generally similar, indicating that the fundamental gluten network structure was maintained despite microstructural changes, resulting in minimal effects on overall quality characteristics.

The volatile compounds measured in this study, including isoamyl acetate, isoamyl alcohol, phenylethyl acetate, phenylethyl alcohol, and ethyl acetate, are common esters and higher alcohols formed during yeast fermentation. The esters isoamyl acetate and ethyl acetate are well known for imparting fruity notes, whereas phenylethyl acetate contributes floral notes [18]. In addition, the higher alcohols isoamyl alcohol and phenylethyl alcohol are associated with alcoholic/malty and floral aromas, respectively, and contribute to the overall aroma profile of fermented foods [18]. Therefore, the differential production of these volatile compounds serves as an important indicator of both yeast fermentation characteristics and bread aroma quality.

The higher levels of isoamyl acetate, phenylethyl alcohol, and ethyl acetate in co-fermented bread than in bread fermented solely with *S. cerevisiae* suggest that co-fermentation may enhance fruity and floral flavor characteristics. In contrast, phenylethyl acetate was detected at the highest level in bread fermented solely with *W. anomalus*. These results indicate that the effect of co-fermentation on volatile compound production varies depending on the specific compound. In particular, ethyl acetate was detected at higher levels in co-fermented bread than in bread fermented solely with *W. anomalus*. This suggests that, whereas ethyl acetate produced by *W. anomalus* alone was not sufficiently retained during baking, both the metabolic interactions between the two yeast species and the structural properties of the dough during co-fermentation may have contributed to the final residual level of ethyl acetate. The ethyl acetate concentration in co-fermented bread with NIYL25845 was below the reported odor threshold (OAV < 1) [39], suggesting that its direct contribution to bread flavor was limited. Nevertheless, ethyl acetate production may vary depending on inoculation conditions (e.g., ratio and timing) and other fermentation parameters; therefore, further studies are needed to optimize co-fermentation conditions for producing high-quality bread.

Co-fermentation with *W. anomalus* NIYL25845 enhanced fruity and floral flavor characteristics while maintaining specific volume and textural properties comparable to those of bread fermented solely with *S. cerevisiae*, indicating its potential usefulness as a baking starter. Previous studies [4, 9, 16, 17] on alcoholic beverages have also reported that co-fermentation of *S. cerevisiae* and *W. anomalus* enhances ester production and aroma complexity. Fan et al. [4] suggested that the increased production of ethyl acetate during co-fermentation may be promoted by metabolic interactions between the two yeast species, whereas Wei et al. [17] reported that ester production varied depending on the inoculation ratio and timing. The present study extends these findings from liquid beverage systems to a solid, baked food matrix, demonstrating that the ester-enhancing effects of *W. anomalus*–*S. cerevisiae* co-fermentation are not confined to alcoholic beverage fermentation. However, unlike alcoholic beverage fermentation, bread fermentation involves dough gas production, dough structure formation, and baking, which collectively influence how these effects are reflected in the final bread quality and aroma characteristics. Notably, the fact that elevated ethyl acetate levels persisted despite thermal volatilization during baking indicates that the metabolic interactions reported in beverage systems can survive a fundamentally different physicochemical process, which represents a key novel finding of this study.

Collectively, these findings suggest that *W. anomalus* NIYL25845 is a promising non-*Saccharomyces* starter for bread fermentation, capable of diversifying flavor profiles without compromising key quality attributes.

## Supplemental Materials

Supplementary data for this paper are available on-line only at http://jmb.or.kr.



## Figures and Tables

**Table 1:** Leavening ability of dough fermented with various yeast strains. (Unit: mL)

Strain	Ratio^*^		
	0:0.5	0:1	0.5:0.5
*S. cerevisiae* Saf-instant	159.69 ± 3.55^d^	260.44 ± 4.84^d^	
*W. anomalus* KCTC27761	89.63 ± 0.76^ab^	180.64 ± 3.15^b^	278.43 ± 4.65^a^
*W. anomalus* NIYL11942	85.18 ± 3.90^a^	174.63 ± 1.18^b^	298.89 ± 0.98^b^
*W. anomalus* NIYL11971	90.24 ± 1.64^b^	151.65 ± 2.74^a^	306.32 ± 4.39^b^
*W. anomalus* NIYL25845	122.94 ± 1.81^c^	203.67 ± 3.67^c^	280.57 ± 3.16^a^

^*^Ratio indicates the inoculation ratio of *Saccharomyces cerevisiae* to *Wickerhamomyces anomalus*. Different superscript letters within the same column indicate statistically significant differences (*p* < 0.05).

**Table 2:** Textural properties of bread fermented with different yeast strains.

Strain	Hardness (N)	Springiness	Cohesiveness	Chewiness (N)
Saf-instant	1.81 ± 0.35^a^	0.85 ± 0.02^a^	0.68 ± 0.01^b^	1.10 ± 0.23^a^
Saf-instant + KCTC27761	1.96 ± 0.18^a^	0.84 ± 0.03^a^	0.69 ± 0.01^b^	1.15 ± 0.10^a^
Saf-instant + NIYL25845	1.88 ± 0.12^a^	0.83 ± 0.01^a^	0.69 ± 0.04^b^	1.21 ± 0.27^a^
KCTC27761	1.98 ± 0.29^a^	0.86 ± 0.03^a^	0.61 ± 0.01^a^	1.11 ± 0.14^a^
NIYL25845	1.77 ± 0.21^a^	0.83 ± 0.02^a^	0.63 ± 0.02^a^	0.99 ± 0.02^a^

Different superscript letters indicate statistically significant differences within the same column (*p* < 0.05).
