# Supplementary material for: Co-Fermentation of Wickerhamomyces anomalus and Saccharomyces cerevisiae in Bread Dough: Effects on Ethyl Acetate Production and Flavor Enhancement
Source: J Microbiol Biotechnol. 2026 Jul 30;36:e2604026. doi: 10.4014/jmb.2604.04026 (PMC13486751; doi:10.4014/jmb.2604.04026)
Supplement: Supplementary file 1 [file jmb-36-e2604026-supple.pdf]

J. Microbiol. Biotechnol. 2026. 36: e2604026
https://doi.org/10.4014/jmb.2604.04026

Supplementary Table and Figures

Table S1. Yeast strains used in this study.

Strain	Name	Source
Saf-instant	*Saccharomyces cerevisiae*	Lesaffre, France
KCTC27761	*Wickerhamomyces anomalus*	Nuruk
MBY1368	*Wickerhamomyces anomalus*	Makgeolli
MBY1373	*Wickerhamomyces anomalus*	Makgeolli
MBY1377	*Wickerhamomyces anomalus*	Makgeolli
MBY/L5183	*Wickerhamomyces anomalus*	Soil
MBY/L5250	*Wickerhamomyces anomalus*	Soil
MBY/L6255	*Wickerhamomyces anomalus*	Apple
NIYL7848	*Wickerhamomyces anomalus*	Makgeolli
NIYL7849	*Wickerhamomyces anomalus*	Makgeolli
NIYL11942	*Wickerhamomyces anomalus*	Soil
NIYL11971	*Wickerhamomyces anomalus*	Soil
NIYL25845	*Wickerhamomyces anomalus*	Nuruk
NIYL30536	*Wickerhamomyces anomalus*	Doenjang
NIYL30823	*Wickerhamomyces anomalus*	Makgeolli
NIYL30826	*Wickerhamomyces anomalus*	Makgeolli
NIYL31017	*Wickerhamomyces anomalus*	Cheonggukjang
NIYL31112	*Wickerhamomyces anomalus*	Soil
NIYL31351	*Wickerhamomyces anomalus*	Soil
NIY1654	*Wickerhamomyces anomalus*	Pear

Fig. S1. Specific growth rates of *Wickerhamomyces anomalus* strains in LD broth at 30°C. Data are presented as means ± standard deviations of three independent experiments. Different letters denote statistically significant differences ($p < 0.05$).

Fig. S2. Gas production by *Wickerhamomyces anomalus* strains in LD broth at 30°C for 6 h. Data are presented as means ± standard deviations of three independent experiments. Different letters denote statistically significant differences ($p < 0.05$).

Fig. S3. Hemolytic activity of *Wickerhamomyces anomalus* strains on Columbia agar supplemented with 5% sheep blood. Cells in the exponential growth phase were harvested, adjusted to an OD_{600} of 0.1, spotted onto plates, and incubated at 30°C for 24 h.

Fig. S1

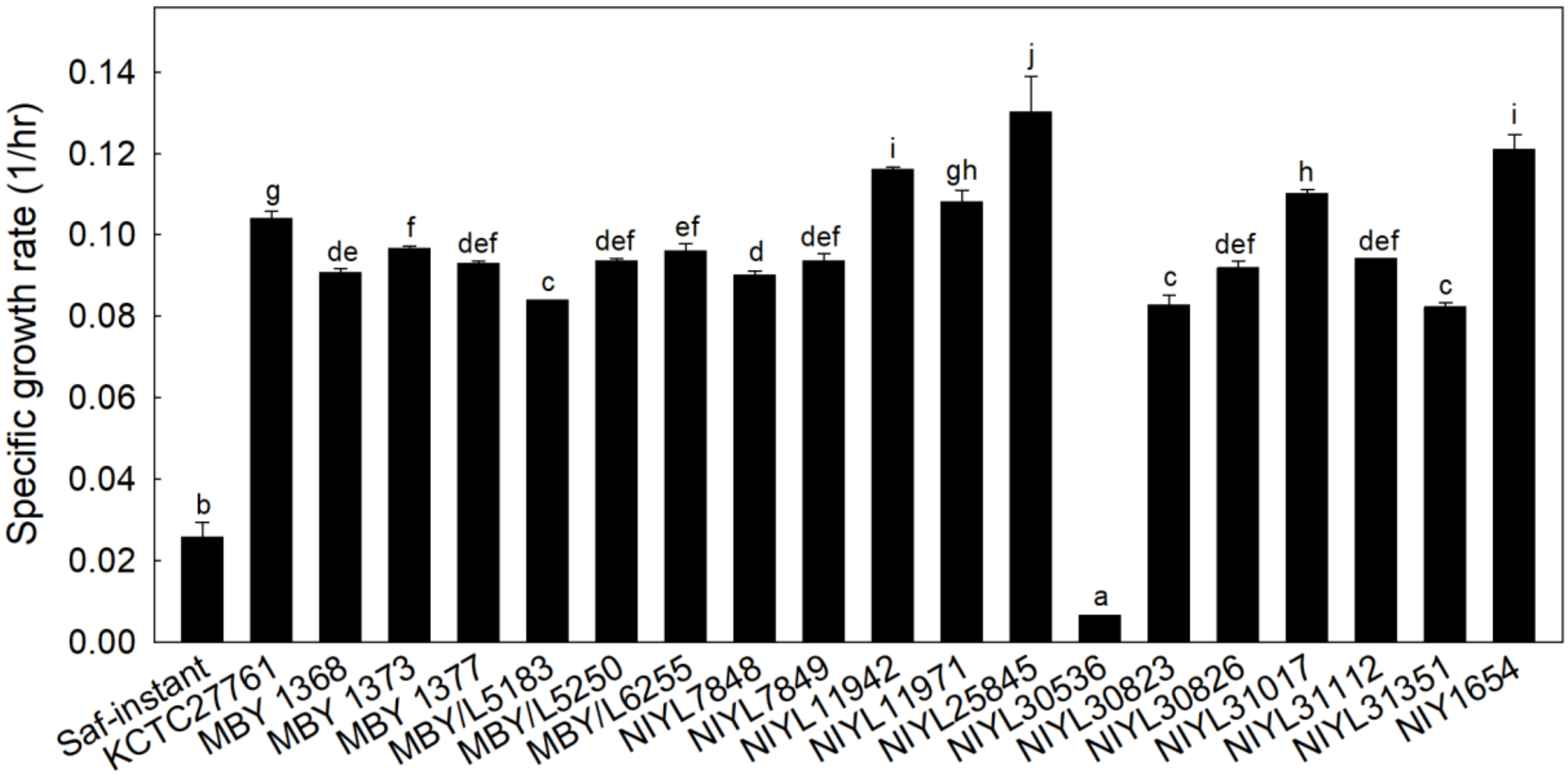
Specific growth rate (1/hr)
0.00
0.02
0.04
0.06
0.08
0.10
0.12
0.14
b
g
de
f
def
c
def
ef
d
def
i
gh
j
a
c
def
h
def
c
i
Saf-instant
KCTC27761
MBY 1368
MBY 1373
MBY 1377
MBY/L5183
MBY/L5250
MBY/L6255
NIYL7848
NIYL7849
NIYL11942
NIYL11971
NIYL25845
NIYL30536
NIYL30823
NIYL30826
NIYL31017
NIYL31112
NIYL31351
NIY1654

Fig. S2

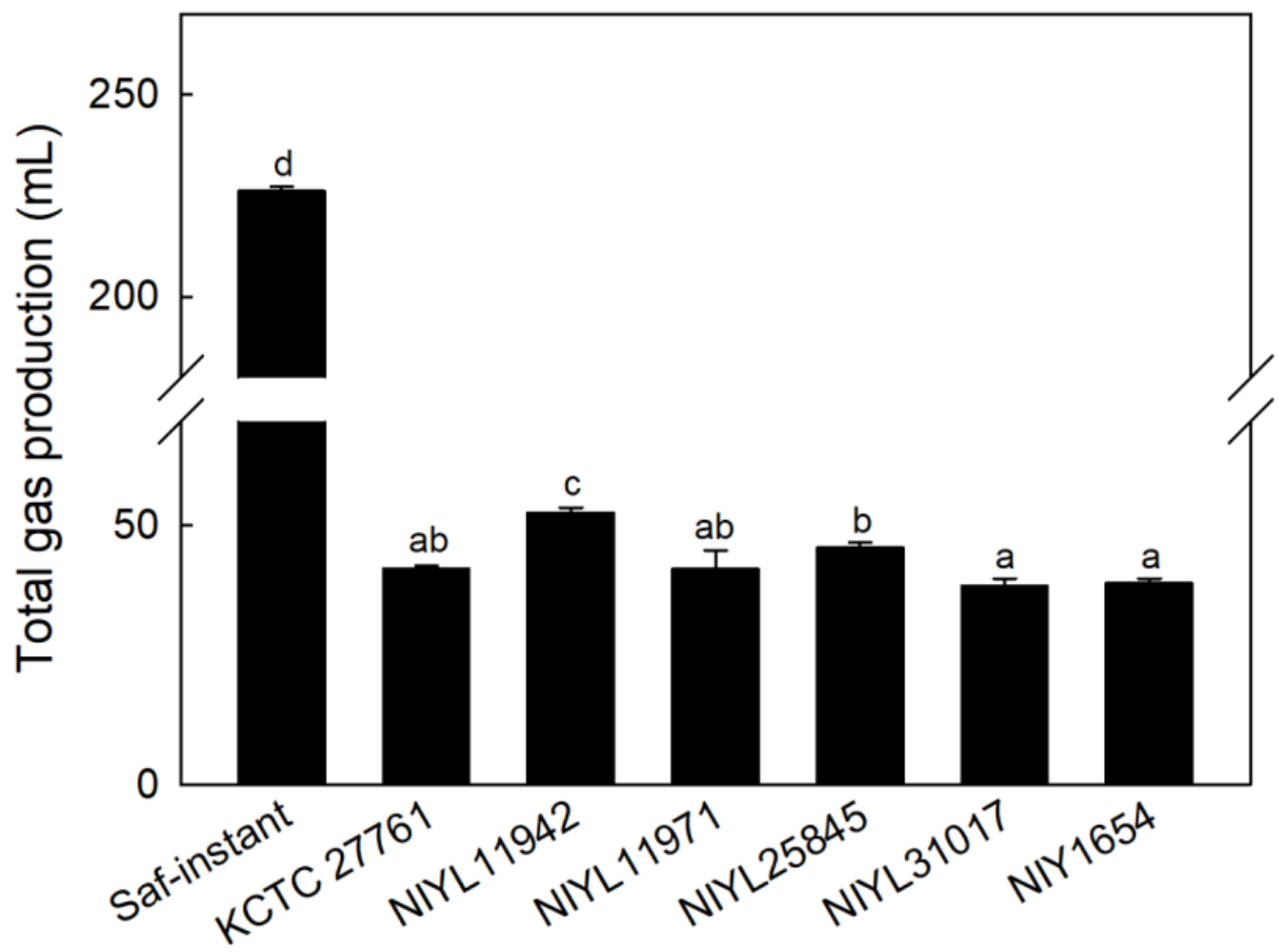
Total gas production (mL)
250
200
50
0
d
ab
c
ab
b
a
a
Saf-instant
KCTC 27761
NIYL11942
NIYL11971
NIYL25845
NIYL31017
NIY1654

Fig. S3

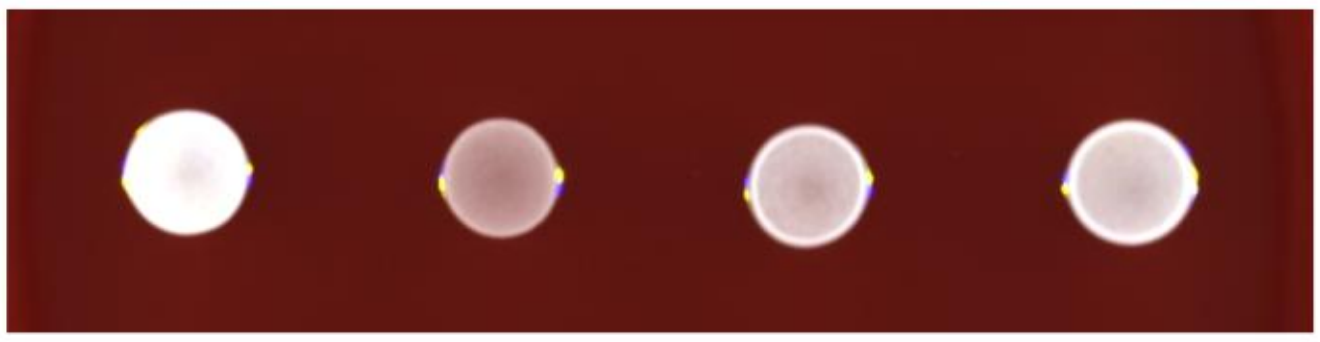

KCTC27761 NIYL11942 NIYL11971 NIYL25845
